# Electrochemical Performance of Deposited LiPON Film/Lithium Electrode in Lithium—Sulfur Batteries

**DOI:** 10.3390/molecules29174202

**Published:** 2024-09-04

**Authors:** Jing Wang, Riwei Xu, Chengzhong Wang, Jinping Xiong

**Affiliations:** 1 College of Basic Education, Beijing Information Technology College, Beijing 100070, China; 2 Beijing Key Laboratory of Electrochemical Process and Technology for Materials, Beijing University of Chemical Technology, Beijing 100029, China

**Keywords:** LiPON film, lithium electrode, interface stability, lithium–sulfur battery

## Abstract

This paper presents a composed lithium phosphate (LiPON) solid electrolyte interface (SEI) film which was coated on a lithium electrode via an electrodeposit method in a lithium–sulfur battery, and the structure of the product was characterized through infrared spectrum (IR) analysis, X-ray photoelectron spectroscopy (XPS), X-ray diffraction (XRD), environment scanning electron microscope (ESEM), etc. Meanwhile, the electrochemical impedance spectrum and the interface stability of the lithium electrode with the LiPON film was analyzed, while the coulomb efficiency and the cycle life of the lithium electrode with the LiPON film in the lithium–sulfur battery were also studied. It was found that this kind of film can effectively inhibit the charge from transferring at the interface between the electrode and the solution, which can produce a more stable interface impedance on the electrode, thereby improving the interface contact with the electrolyte, and effectively improve the discharge performance, cycle life, and the coulomb efficiency of the lithium–sulfur battery. This is of great significance for the further development of solid electrolyte facial mask technology for lithium–sulfur batteries.

## 1. Introduction

Lithium–sulfur batteries (LSBs) are considered one of the most promising candidates for next-generation energy storage owing to their large energy density. Tremendous effort has been dedicated to overcoming the essential problems of LSBs to facilitate their commercialization, such as polysulfide shuttling and dendritic lithium growth [1]. Nevertheless, due to the instability of the SEI film of lithium metal anodes, the dendrites of lithium negative electrodes are constantly generated and grown in the course of the cycle process, which greatly limits the performance of lithium anodes [2,3]. Domestic and foreign scholars have been exploring strategies and approaches to address these challenges: exploring new solvent and electrolyte systems [4,5], developing new lithium salts and high–performance additives [6,7], and so on. However, a LiPON film can easily overcome the above difficulties, with its high ionic conductivity, superior mechanical flexibility, and excellent electrochemical stability [8,9], which provides effective protection for lithium–sulfur batteries, significantly improving their lifespan, safety, and performance [10]. Bates et al. [11] prepared the earliest solid electrolyte of oxynitride glass—LiPON film—by sputtering Li_3_PO_4_ target in a high-purity N_2_ atmosphere via magnetron sputtering technology. The conductivity of the product reached 2 × 10^−6^ S/cm at room temperature. Suzuki et al. [12] prepared a LiPON electrolyte membrane with a Li_2_O + Li_3_PO_4_ composite target, and the conductivity of the product reached 6.4 × 10^−6^ S/cm at room temperature. Lee et al. [13] synthesized a LiSiPON amorphous electrolyte with a lithium phosphate–lithium silicate (xLi_4_SiO_4_ − (1 − x) Li_3_PO_4_) composite target via a magnetron sputtering method. The ionic conductivity test results illustrated that the activation energy of Li^+^ migration decreased with the increase of silicon content in the system, which enhanced the Li^+^ conductivity of the system to 1.24 × 10^−5^ S/cm at room temperature. Due to its good electrochemical stability, LiPON films are chiefly studied for use in solid-state electrolytes. Currently, there is little research on the specific effects and applicability of LiPON film in liquid electrolytes, and further exploration and verification through experiments are still needed.

In this paper, LiPON films were prepared via magnetron sputtering deposition, and their properties and structures were characterized by infrared spectrum analysis, XPS, X-ray diffraction, SEM, an oxygen–nitrogen analyzer, etc. At the same time, the electrochemical impedance and lithium-ion conductivity of the LiPON films were studied, and the protective effect of LiPON films on lithium–sulfur battery electrodes in organic electrolyte systems was examined as well. The results showed that the electrodeposited LiPON film could effectively improve the coulombic efficiency and cycle life of lithium–sulfur batteries in liquid electrolytes, providing a new idea to improve the safety and electrochemical performance of lithium-ion batteries.

## 2. Result and Discussion

### 2.1. The Properties of LiPON Film

**Figure 1:**
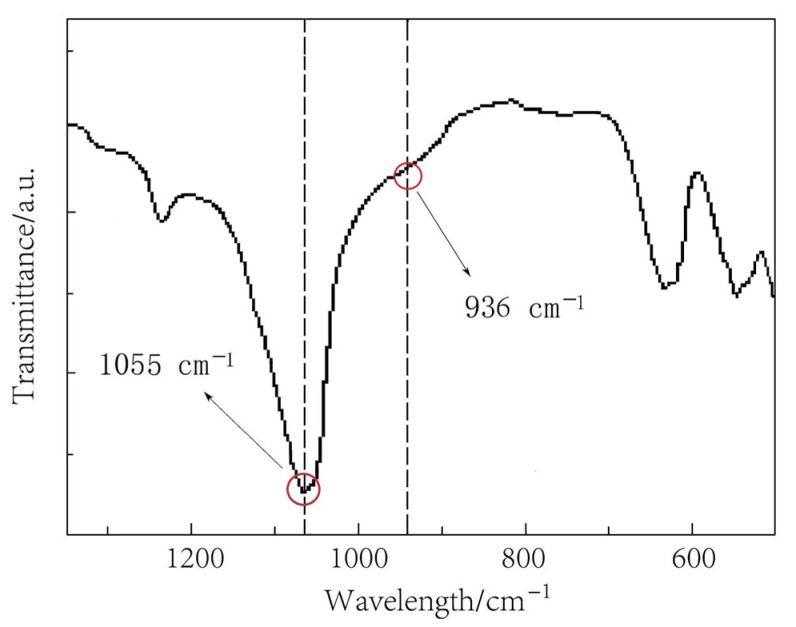
The FTIR spectrum of the prepared LiPON film.

**Figure 2:**
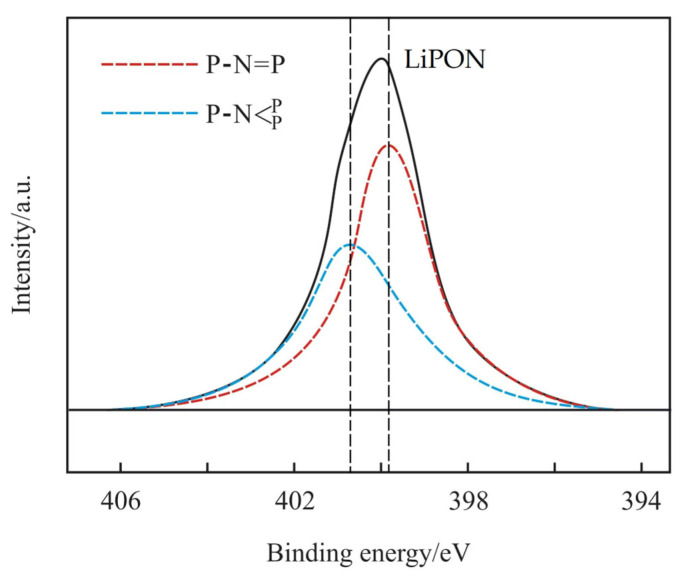
The XPS N1s spectrum of the prepared LiPON film.

The FTIR spectrum of the prepared LiPON film is shown in Figure 1. The peak at 1055 cm^−^^1^ was attributed to the symmetric vibration of PO_3_ structure, and the peak near 936 cm^−^^1^ referred to the asymmetric vibration of the P-O-P structure [14]. The results showed that a new structure was formed. In order to prove this further, an XPS N1s spectrum was used for analysis, as shown in Figure 2. Through fitting, the peak was resolved into two peaks, one at 400.68 eV corresponding to the 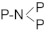 structure, and the other at 399.73 eV corresponding to the P-N = P structure, which was consistent with the reference results [15]. It was confirmed that chemical bonds were formed, as was the result in the above FTIR results, and that this helped to increase the mesh structure in the film, thereby improving the ion conductivity.

**Figure 3:**
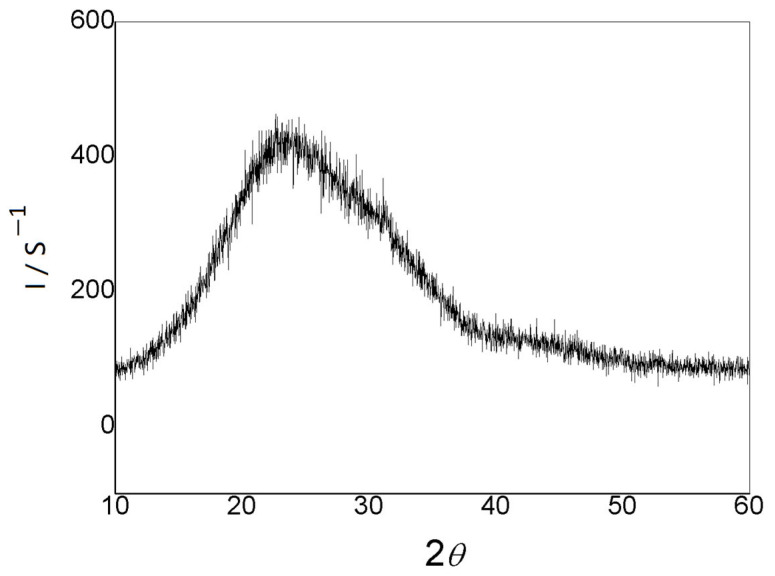
XRD illustrative pattern of the prepared LiPON film.

The XRD structure of the LiPON film is shown in Figure 3, and no obvious diffraction peaks were observed. Instead, a wide envelope appeared, indicating that the film structure was amorphous. Compared to the crystalline phase, the LiPON film with a certain structure acted as an ion conductor without grain interfaces, and its composition could continuously change over a wide range. Therefore, the diffusion pathway and physical properties of ions were isotropic, resulting in a decrease in interface impedance and ensuring ions could pass through the particle interfaces more easily, which led to an increase in conductivity as well [16,17].

**Figure 4:**
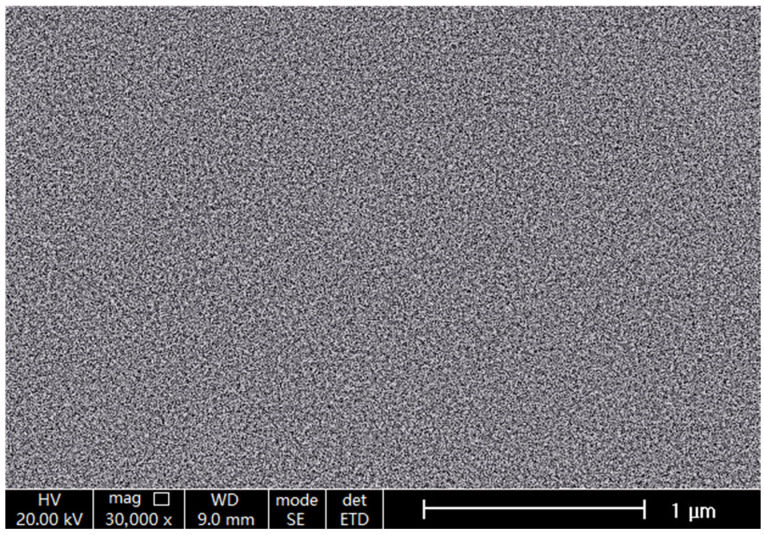
ESEM image of the prepared LiPON film.

Figure 4 shows the ESEM image of the prepared LiPON film. It can be seen that the surface of LiPON film did not have an ordered arrangement of particle structures, nor did it exhibit any crystalline characteristics. This proved that the film possessed an amorphous structure, which was consistent with the XRD results above. Further, the product structure was obviously tight and the surface was smooth, without defects such as holes, cracks, overlapping protrusions, or blank areas. This film structure not only prevented the electrolyte from reacting with metallic lithium, but also prevented short circuits between the positive and negative electrodes inside the battery; at the same time, it can also provide good conductivity and a good contact interface between the positive and negative electrodes, and maintain the function of the electrolyte.

### 2.2. Electrochemical Measurements

**Figure 5:**
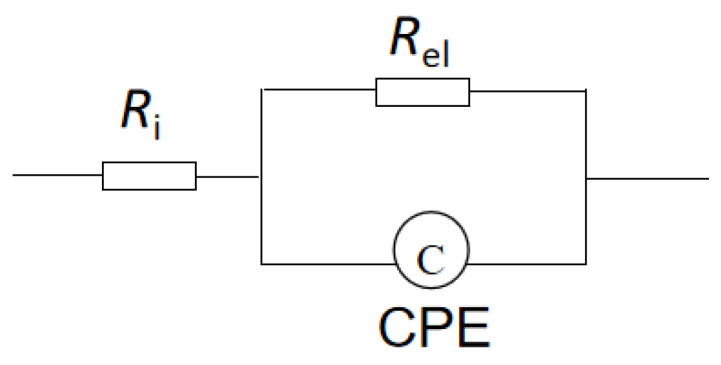
The Randles circuit of a lithium electrode.

**Figure 6:**
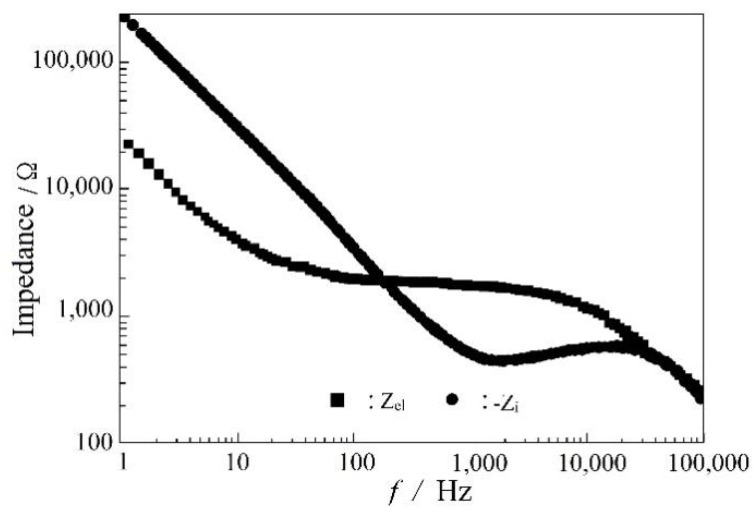
The Bode plot of Al/LiPON/Al.

Figure 5 is an equivalent circuit diagram of this lithium electrode. R_i_ is the bulk impedance of the solution; R_e_l is the interface resistance of the lithium electrode, and CPE is the constant phase element. Through the analysis of this equivalent circuit, the characteristic information about the lithium electrode interface can be obtained, as shown in the Bode plot of a sandwich structure composed of LiPON films (Al/LiPON/Al; Figure 6). In this model circuit, the low-frequency part of the interface impedance played the leading role, and in the high-frequency part, the ion conduction in the film medium and the polarization of bound charges played a major role accordingly.

**Figure 7:**
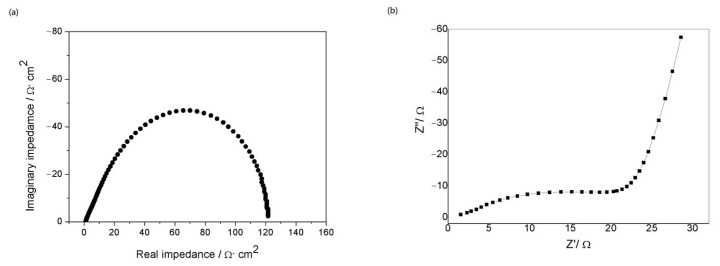
(**a**) the EIS of a metallic lithium electrode in organic electrolyte; (**b**) Nyquist plot of sandwich structure of Al/LiPON/Al.

The electrochemical impedance spectrum (EIS) of a metallic lithium electrode in organic electrolyte—1M LiTFSI/DOL: DME (1:1)—can be seen in Figure 7a. The test results were consistent with the impedance studies of lithium electrodes in the literature [18], and the spectrum showed a semicircle. While the Nyquist plot of Al/LiPON/Al in Figure 7b almost accords with the impedance graph of a typical solid electrolyte film as well, the middle–high frequency part does not appear as a complete semicircle. The main reason might be that there existed a layer of Al_2_O_3_ oxide film on the LiPON surface, which prevented direct contact between the electrolyte and the electrode material, changing the path of ion conduction on the electrode surface and resulting in the alteration in the characteristic frequency range of EIS [19].

The fitting of the equivalent circuit model (Figure 5) to the AC impedance spectrum indicated that the diameter of the semicircle corresponded to the bulk resistance Z of the solid electrolyte, and the ion conductivity of the prepared LiPON electrolyte film was calculated to be 2.4 × 10^−7^ S/cm via the formula σ = d/(ZA) (where d referred to the thickness of the solid electrolyte and A was the effective cross-sectional area of the device).

**Figure 8:**
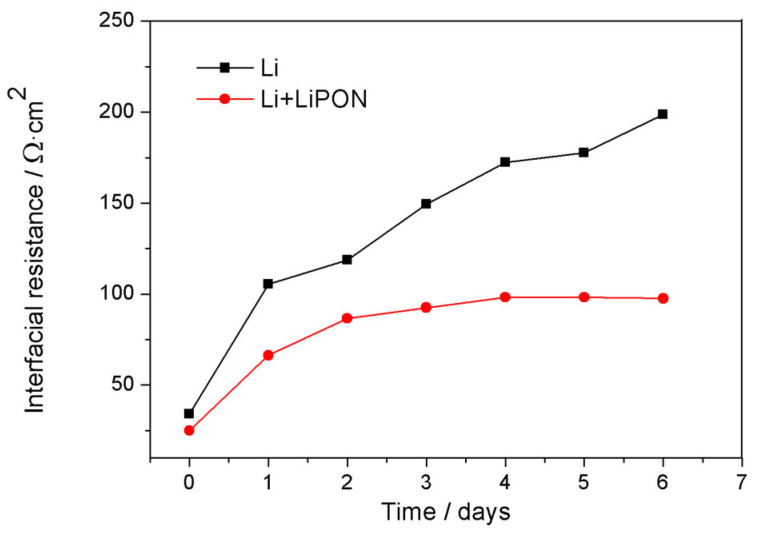
Changes of impedance of different lithium electrodes with soaking time.

Figure 8 showed the electrochemical impedance changes of a bare lithium electrode and lithium electrode protected by LiPON film when they were placed in 1 M LiTFSI/DOL:DME (1:1) electrolyte. From the experimental results, the impedance of the bare lithium electrode increased with the soaking time. This is because, when the electrode was initially immersed in the electrolyte, the SEI interface membranes were formed on the surface of lithium, and the film continued to grow, leading to the continuous increase of interface resistance. Compared with lithium electrodes protected by a LiPON film, lithium electrodes can achieve steady-state interface impedance faster, as LiPON film can act as the passivation layers which covered around the surface of lithium electrode and formed a more stable SEI film, thereby reducing interface resistance and reactions, and maintaining long-term interface stability [20,21,22].

In order to further verify the improvement effect of LiPON film protection on lithium electrode in lithium–sulfur battery, the performance of the experimental battery was also investigated.

**Figure 9:**
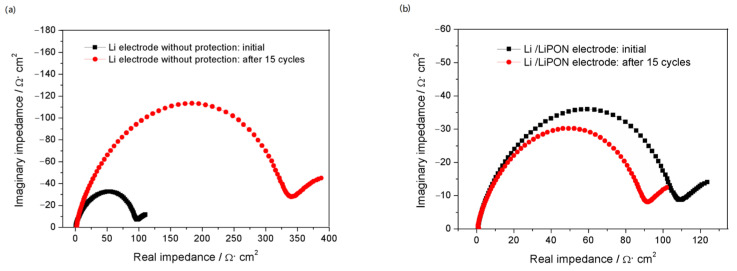
(**a**) The EIS of Li electrode without protection in charging and discharging process; (**b**) the EIS of Li electrode with LiPON protection in charging and discharging process.

**Figure 10:**
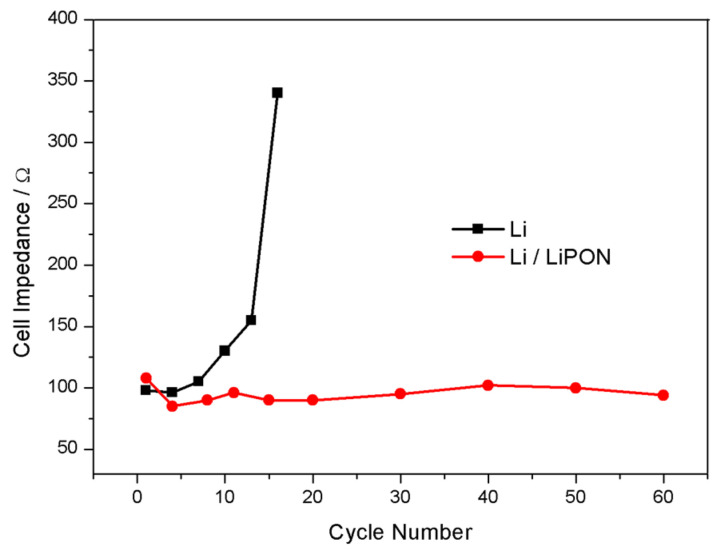
Changes of impedance of batteries composed of different lithium electrodes with cycle times.

Figure 9 showed the EIS of the lithium electrode with and without LiPON film protection during charge and discharge processes. When there was no LiPON film protection, the interface impedance of the lithium electrode increased significantly with the increase of cycle number, reaching nearly four times the initial state after 15 cycles, while the interface impedance of the lithium electrode with LiPON film remained almost unchanged, or even lower than the initial state. From the comparison results in Figure 10, it can be seen more clearly that, during the cycling process, the interface impedance of the lithium electrode without the LiPON film increased sharply, while the interface impedance of lithium electrodes with the LiPON film was relatively stable, which can even last up to 60 times. This was mainly because the lithium electrodes without the LiPON film’s protection had very unstable surfaces during the charge and discharge processes, which led to the uncontrolled growth of the SEI films, increased the resistance of lithium ion transport, and caused a continuous increase in interface impedance, thereby affecting the performance and lifespan of the lithium–sulfur battery [23,24].

When there was a LiPON film on the negative electrode of the lithium–sufur battery, the extremely active lithium reacted with the anions in the electrolyte, and the reaction products (insoluble) were deposited on the surface of lithium to form a passivation film that was thick enough to prevent electrons from passing through. The SEI film continued growing until it had sufficient thickness and compactness to prevent the co-insertion of solvent molecules, which ensured the stability of electrode circulation. The reaction mechanism can be seen as follows [25,26]:

PC + 2 e^−^ + 2Li^+^ →CH_3_CH(OCO_2_Li) CH_2_ (OCO_2_Li) ↓ + CH_3_CH = CH_2_ ↑

2EC + 2 e^−^ + 2Li^+^ →(CH_2_OCO_2_Li)_2_ ↓ + CH_2_ = CH_2_ ↑

Therefore, lithium electrodes protected by a LiPON film exhibited lower and more stable interface impedance. It once again proved that LiPON film can effectively improve lithium ion transport, can reduce adverse reactions between electrode materials and electrolytes, can slow down the excessive growth of passivation layers on the surface of lithium electrodes, and can help to form smoother lithium deposition. This would not only reduce the dendrite growth, but also maintain low interface impedance, thereby improving the electrochemical performance of lithium–sulfur batteries.

**Figure 11:**
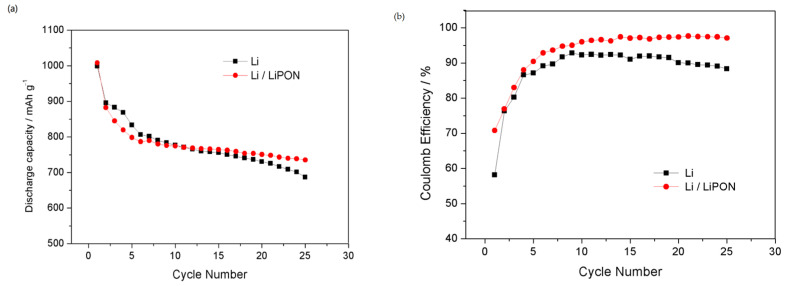
(**a**) Cycle performance of different lithium electrodes in the charge–discharge cycles; (**b**) coulomb efficiency of different lithium electrodes in the charge–discharge cycles.

Figure 11 shows the battery cycle life and the coulombic efficiency of lithium electrodes protected by a LiPON film and bare lithium electrodes in lithium–sulfur batteries. The results showed that the cycling stability of the lithium electrode protected by a LiPON film was significantly improved, with an initial discharge capacity of 1008.4 mAh/g. After 40 cycles, it still maintained a discharge capacity of 735.2 mAh/g, 27.1% lower than the initial discharge capacity; whereas the discharge capacity of the bare lithium electrode decreased by 31.2% after 25 cycles. The coulombic efficiency of lithium electrodes protected by a LiPON film can still stay at 97.1% after 25 cycles, significantly higher than that of bare lithium electrodes. LiPON, as a dense protective film, helped to stabilize the structure of the lithium electrode surface and suppressed the shuttle effect of lithium polysulfides; at the same time, it can effectively prevent side reactions between lithium electrodes and electrolytes, forming a more stable interface, inhibiting the growth of lithium dendrites, and thus improving the cycle life and coulombic efficiency of lithium–sulfur batteries [25]. However, the lithium electrode without LiPON film protection was constantly broken and repaired due to the fragility of the surface facial mask, resulting in an increase in the thickness of the interface membrane, which not only enhanced the internal impedance of the battery, but also led to the continuous loss of the lithium electrode material. The generation of lithium dendrites and “dead lithium” acutely accelerated the attenuation of the battery performance [26].

**Figure 12:**
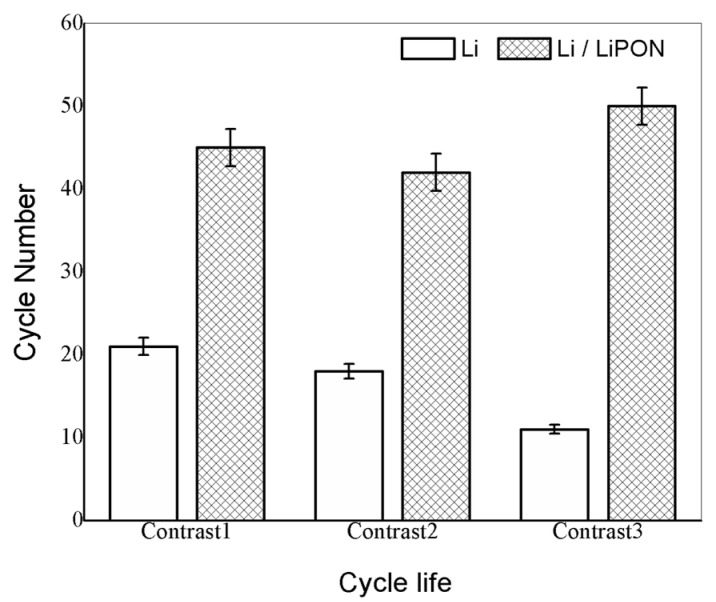
Comparison of cycle life of two groups of batteries with different lithium electrodes.

On the basis of the above experiment, we further conducted multiple experiments to compare the cycle life of lithium electrodes without LiPON film protection and those with LiPON film protection. From the results in Figure 12, it can be seen that the cycle life of lithium electrodes with a LiPON film basically exceeded 40 cycles, while the unprotected lithium electrodes quickly terminated their cycle life. This was because lithium electrodes would undergo volume changes during the charging and discharging process. Without a protective film, the electrode surface would become uneven and lithium ions would form uneven deposits, making it easier to form lithium dendrites, which resulted in the loss of active materials and the destruction of electrode structures, ultimately leading to a gradual decline in the available capacity of the battery [27].

In this way, the existence of the LiPON film provided an excellent physical barrier which can effectively improve the stability of lithium electrodes and extend the cycle life of lithium–sulfur batteries. The cycle life error of lithium batteries was about 5%. The main reason was that changes of transistor temperature during the testing process would cause variations in the accuracy of the constant current source, which would affect the accuracy of battery life [28].

## 3. Materials and Methods

### 3.1. Material Preparation

In this experiment, LiPON films were prepared via radio frequency magnetron sputtering, with Li_3_PO_4_ (purity over 99.99%) as the sputtering target material, high-purity N^2^ as the sputtering gas, and ultrasonic-cleaned quartz glass as the substrate. The sputtering chamber had been evacuated to 5 × 10^−4^ Pa, and the working pressure was set to 0.5 Pa, while the RF power was adjusted to 100 W. After the distance between the target and the substrate had been adjusted, LiPON was sputtered. The Al electrodes deposited on both sides of the LiPON film were used as blocking electrodes to form an Al/LiPON/Al sandwich structure.

In the organic electrolyte system (1 M LiTFSI (DOL/DME = 1:1)), a nickel electrode deposited with a LiPON film was used as the working electrode, and a lithium electrode was used as the counter electrode. By depositing a certain amount of lithium on the working electrode, a lithium electrode coated with a LiPON film was obtained.

### 3.2. Characterizations

Fourier-transform infrared spectroscopy (FTIR, Nicolet-60 SXB, Thermo Fisher Scientific molecular spectroscopy, Shanghai, China) and X-ray photoelectron spectroscopy (XPS, Thermo esca lab 250Xi, Suzhou Sains Instrument Co., Ltd., Suzhou, China) were used to determine the elemental composition of LiPON. An X-ray diffractometer (XRDynamic 500/Primux 3000, Anton Paar (Shanghai) Trading Co., Ltd., Shanghai, China) was used to study the structure of the prepared LiPON film, and the surface morphology of the LiPON film was observed by environmental scanning electron microscope (PRISMA, Thermo Fisher Scientific (China) Co., Ltd., Shanghai, China).

The electrochemical impedance spectrum and cyclic performance of the product were measured through an electrochemical workstation (Arbin, Suzhou Tianyi Kechuang Electromechanical Technology Co., Ltd., Suzhou, China; with the test frequency range 0.1 MHz–1 MHz, the disturbance amplitude 10 mV, and the constant temperature guaranteed at 25 °C), and the lithium ion conductivity of the LiPON film was calculated [29].

### 3.3. Electrochemical Measurement

A button cell was assembled for further experiments with a carbon black–sulfur composite material as the positive electrode, lithium electrodes coated with the LiPON film and bare lithium electrodes as the negative electrodes (comparative tests), and a LiTFSI (DOL/DME = 1:1) solution as the electrolyte.

Electrochemical impedance spectroscopy was adopted to test the change of interface impedance with the soaking time of the lithium electrode coated with the LiPON film and that of the bare lithium electrode without the LiPON film separately, so as to verify the interface stability.

The Arbin electrochemical workstation was used to conduct the cyclic performance tests on lithium electrodes with and without LiPON film protection, under the condition of an electric capacity of 0.5 mAh/cm^2^. The cycle life and the coulombic efficiency of the batteries were measured under the two different conditions during the experiment. At the same time, the battery impedance was tested every three cycles to record the change patterns during the cycle.

## 4. Conclusions

In this study, a LiPON film was successfully coated on the surface of metal lithium electrodes by RF magnetron sputtering technology, and its structure and composition were characterized via IR, XPS, XRD, and ESEM. Through the impedance analysis of the Al/LiPON/Al sandwich structure, the ionic conductivity of the LiPON film was calculated to be 2.4 × 10^−7^ S/cm. At the same time, it was found that the impedance of lithium electrode protected by the LiPON film changed little with the increase of the soaking time in the organic solution. In the lithium–sulfur battery, the impedance of the lithium electrode protected by the LiPON film was relatively stable with the increase of the cycle number, and could maintain a longer number of cycles; after 40 cycles, the specific energy of the battery remained 735.2 mAh/g, which was 27.1% lower than the initial state; whereas the specific energy of the electrode without the LiPON film decreased by 31.2%, and the cycle was soon terminated. These experimental results showed that, because of the electrochemical stability, high lithium-ion conductivity, and good interface compatibility, a LiPON film can reduce the adverse reactions between electrode and electrolyte, and improve the stability of the electrode/electrolyte interface. Especially in a lithium–sulfur battery system, the application of a LiPON film can also effectively block the shuttle of polysulfides and prevent the growth of lithium dendrites, improving the coulomb efficiency and cycle stability of the battery, and prolonging the battery life. It played an important role in improving the overall performance of lithium–sulfur battery.

## Data Availability

Dataset available on request from the authors.
